# Access to cardiovascular medicines in low- and middle-income countries: a mini review

**DOI:** 10.1186/s41256-023-00301-6

**Published:** 2023-05-23

**Authors:** Mark Amankwa Harrison, Afia Frimpomaa Asare Marfo, Augustine Annan, Daniel Nii Amoo Ankrah

**Affiliations:** 1 Pharmacy Department, Korle Bu Teaching Hospital, Korle Bu, P.O. Box 77, Accra, Ghana; 2 Department of Pharmacy Practice, Faculty of Pharmacy and Pharmaceutical Sciences, College of Health Sciences, Kwame Nkrumah University of Science and Technology, Kumasi, Ghana

**Keywords:** Cardiovascular medicines, Access, Low- and middle-income countries, Affordability, Availability

## Abstract

**Background:**

Many cardiovascular (CV) medicines are required for long term. However, with their limited resources, low- and middle-income countries (LMICs) may have challenges with access to cardiovascular medicines. The aim of this review was to provide a summary of available evidence on access to cardiovascular medicines in LMICs.

**Methods:**

We searched PubMed and Google scholar for English language articles on access to cardiovascular medicines for the period 2010–2022. We also searched for articles reporting measures for challenges in access to CV medicines from 2007 to 2022. Studies conducted in LMICs, and reporting availability and affordability were included for review. We also reviewed studies reporting affordability or availability using the World Health Organisation/Health Action International (WHO/HAI) method. Levels of affordability and availability were compared.

**Results:**

Eleven articles met the inclusion criteria for review on availability and affordability. Although availability appears to have improved, many countries did not meet the availability target of 80%. Between economies and within countries, there are equity gaps in access to CV medicines. Availability is lower in public health facilities than private facilities. Seven out of 11 studies reported availability less than 80%. Eight studies which investigated availability in the public sector reported less than 80% availability. Overall, CV medicines, especially combined treatments are not affordable in the majority of countries. Simultaneous achievement of availability and affordability target is low. In the studies reviewed, less than 1–53.5 days wages were required to purchase one month supply of CV medicines. Failure to meet affordability was 9–75%. Five studies showed that, on average 1.6 days’ wages of the Lowest-Paid Government Worker (LPGW) was required to purchase generic CV medicines in the public sector. Efficient forecasting and procurement, increased public financing and policies to improve generic use, among others are measures for improving availability and affordability.

**Conclusions:**

Significant gaps exist in access to cardiovascular medicines in LMICs, and in many low—and lower middle—income countries access to cardiovascular medicines is low. To improve access and achieve the Global Action Plan on non-communicable diseases in these countries, policy interventions must be urgently instituted.

## Introduction

Cardiovascular disease (CVD) is the leading cause of death globally [1]. Low- and middle-income countries (LMICs) bear a growing burden of mortality attributable to CVD. Over three quarters of deaths resulting from CVD occurred in LMICs [1]. A recent WHO report shows that while Africa achieved significant reductions in morbidity and mortality rates of HIV/AIDS and Malaria, CVD (ischaemic heart disease and stroke) recorded very marginal reductions [2].

Sustained availability and accessibility of medicines are critical for the prevention and management of CVD as many cardiovascular (CV) drugs are required for long term. While the Sustainable Development Goal 3 urges countries to ensure access to safe, effective, quality and affordable essential medicines for all, LMIC are resource limited [3]. As part of the Global Action Plan (GAP) for Non-communicable diseases (NCD), the World Health Organisation (WHO) has set a national target of 80% availability for affordable essential medicines such as generics required to treat major NCDs in both public and private facilities to be achieved by 2025 [4]. The WHO created the Essential Medicines List (EML) aimed at ensuring the availability of medicines such as cardiovascular (CV) medicines within the public health system of such countries [5]. Antihypertensive drugs, lipid-lowering drugs, antiplatelets, medicines for heart failure and cardiac arrhythmia and anti-anginal drugs are found in the WHO model list of CV medicines [5]. Antihypertensive drugs, lipid lowering drugs and antiplatelets are among the most frequently used CV medicines. While EMLs are expected to ensure the availability of medicines in LMICs, several factors may stifle the availability of CV medicines in such countries. Also, while CV medicines may be available in the health system, they may not be adequately accessible and affordable to patients at the point of care.

Gaps in access to cardiovascular medicines exist even in high income countries, and middle- and low-income countries have resource challenges. Therefore, access to CV medicines in LMICs needs to be evaluated to inform current knowledge as well as policy directions that can improve access to cardiovascular medicines and reduce CV-related mortality burden. While facility-based and national surveys have been conducted in resource-limited settings, reviews that provide a current overview of the cardiovascular medicine access situation are needed. The aim of this review was to provide a summary of available evidence on access to cardiovascular medicines in low- and middle-income countries.

## Methods

### Research design

We conducted a mini-review of peer-reviewed articles in literature. It was a review of surveys/cross sectional studies on access to cardiovascular medicines using a systematic search of literature**.** Mini-reviews are reviews that summarize the most salient concepts of a topic and still ensure that the most current and relevant findings are reported.

### Literature search

We searched PubMed and Google scholar for articles on access to cardiovascular medicines. We used the search terms “cardiovascular medicines”, affordability”, “availability”, “access”, and “antihypertensive medicines”. We searched for English language articles from 2010 to 2022. We crosschecked PubMed and Google articles to avoid duplication. We also searched for articles reporting measures for improving access to cardiovascular medicines from 2007 to 2022 using the terms “medicines access”, “affordability and availability measures” and “cardiovascular medicines access”.

### Inclusion criteria

Studies conducted in low- and middle-income countries were included. We reviewed studies reporting availability and affordability of cardiovascular medicines. We reviewed studies reporting affordability or availability using the WHO/HAI method. Surveys conducted at the population level or national surveys and facility level surveys which reported antihypertensive medicines, statins or antiplatelet or combination of any two or three were reviewed.

### Screening process and data extraction

We selected articles based on an apriori criteria. Abstracts and titles were screened before full text articles. The articles were reviewed by two experts. A data abstraction form developed by the principal investigator and one co-investigator was used to document abstract article details. We retrieved data on Author and year of publication, study design, study setting, sample size, type of CV drug (Antihypertensive, statins and anti-platelets) studied, number of CV medicines and summary of findings. Data abstraction was done by the principal investigator.

### Evidence synthesis

The data abstracted from articles which met the inclusion criteria were reviewed by the principal investigator and a co-investigator and analysed by comparing results from the studies. Trends, similarity and contradiction in findings were determined. Levels of affordability and availability were compared between studies as well as between groups or geographic settings in the same study. The WHO/HAI definition of affordability and availability was used to review affordability and availability for studies that used the WHO/HAI method to measure affordability and availability.WHO/HAI reports availability of individual medicines as the percentage (%) of medicine outlets or surveyed facilities in which the medicine was available on the day data was collected. Average % availability across all medicines can also be estimated. Affordability is determined using the daily wage of the lowest-paid unskilled government worker. Affordability is the number of days’ wages needed to purchase a selected course of treatment for common acute and chronic conditions. For chronic conditions the WHO/HAI defines affordability as number of days’ wages of the lowest paid government worker needed to purchase one month supply of medicines. For other studies the catastrophic health expenditure method and the proportion of patients unable to afford their medicines was used. The catastrophic health expenditure method classifies medicines as affordable if patients spend less than 20% of household income to purchase one month supply of CV medication. Availability was defined as the proportion of medicines studied that were available for studies reporting proportion of medicines available and proportion of facilities with medicine available for studies reporting proportion of facilities with availability. Medicines were classified as available if availability was 80% or more. The articles were not rated for risk of bias.

### Dimensions of access to medicines

The WHO has defined access to medicines as “having medicines continuously available and affordable at public or private health facilities or medicine outlets that are within one hour’s walk of the population” [6]. Access to medicines has been described in five dimensions namely availability, affordability, accessibility, acceptability, and quality of medicines [7]. These dimensions have been described by Wirtz et al. [7]. However, most studies have focused on availability and affordability as measures of access.

## Results

**Fig. 1:**
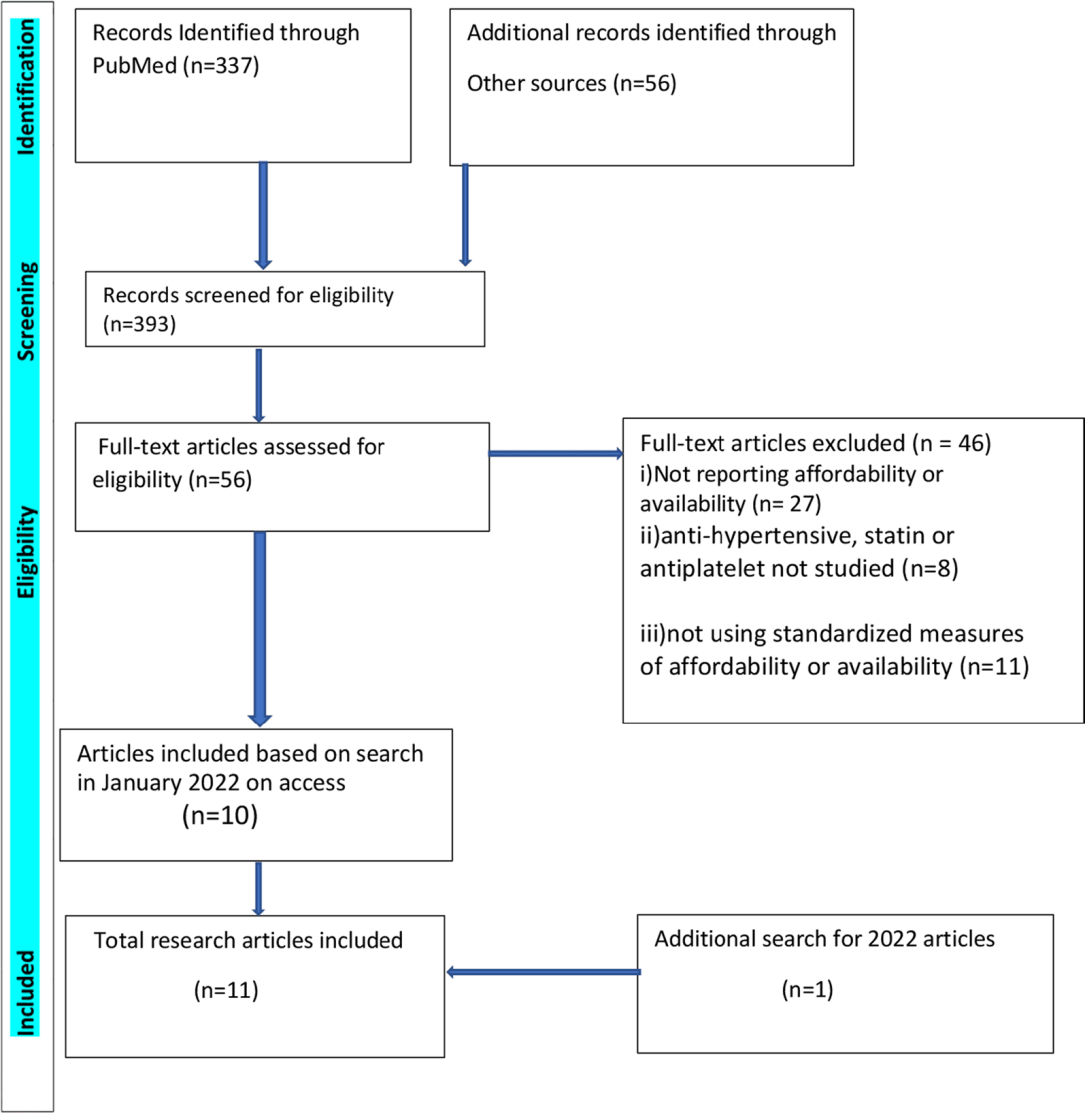
PRISMA flow diagram for the literature search on access to cardiovascular medicines

The total number of articles that were screened for eligibility was 393. As part of this total, 337 articles were identified from PubMed, while 56 were identified from Google Scholar. The number of full text articles assessed for eligibility was 56. Ten full text articles met the inclusion criteria from the search in January 2022. An additional search for articles published in 2022 yielded one additional article. Eleven full text articles were finally included for analysis (Fig. 1).

### The access situation in low- and middle-income countries

Access to CV medicines is a challenge in many LMICs. Between economies and within countries there are equity gaps in access to CV medicines in the health system. In settings or facilities where CV medicines are available, affordability is often a challenge. In settings or facilities where medicines are affordable or supplied free, availability is often a challenge [8–10]. Seven out of 11 studies reported availability less than 80%. Eight studies which investigated availability in the public sector reported less than 80% availability of CV medicines. Attaei et al. have showed that many communities in low- and middle-income countries do not have access to anti-hypertensive medicines [8].

In many low- and middle- income countries, availability of cardiovascular medicines is low. Although availability of CV medicines appears to have improved over the years, it is still sub-optimal in many countries. The availability of CV medicines was 14.4% to 20.8% for generics in the public sector and 52.3% to 60.1% for generics in the private sector in LMICs in an earlier study [11]. In another earlier study the average availability of selected cardiovascular medicines was between 6.3% and 53% in the public sector [12]. Recent studies show low availability of CV medicines. In one study, average availability was 44% [8] in LMICs, and in another study availability was 54% in low and lower-middle income countries (LLMICs) and 60% in upper-middle income countries (UMICs) [13]. A study by Ewen et al. reported that in LICs, median generic CV drug availability was 40.2% and 59.1% in the public and private sectors, respectively. Overall generic availability was 54.6% and 65.7% in lower-middle income countries and 56.7% and 76.7% in UMICs in the public and private sectors, respectively [9]. In a more recent study by Chow et al. published in 2020, the availability of all three types of cardiovascular medicines (blood pressure lowering medicines, anti-platelets and statins) was 50%, 62.8% and 87.2% respectively [14]. Another recent study in the Middle East shows cardiovascular medicine availability of 36.6–52.5% [15]. In East Africa, average availability of cardiovascular medicines in one study was 55.7%, while in another study antihypertensive medicine availability was 0–28.5% and heart failure medication availability was 0.5–49.5% [16, 17]. A recent study in West-Central Africa reported availability of 25.3–49.2% [14]. In 2022, a large study reported 19.03–76.9% availability for LPG in the public sector and 41.1–80.49% availability of LPG in the private sector [18]. Availability of cardiovascular medicines is lower in public health facilities than private facilities in LMICs [9, 13, 15, 18]. Branded medicines are less available than generic medicines. The average availability of branded CV medicines in LLMICs was 20% in the public sector and 34% in the private sector [11]. In a study by Van Mourik et al., CV medicines had an average availability of 26.3% for the lowest price generics in the public sector and 57.3% in the private sector [11]. Husain et al. reported similar results with a generic CV drug availability of 55% and 67% in public and private sectors respectively [13]. Availability of CV medicines is higher in urban than rural areas [8].

CV medicines overall were not affordable in the majority of countries, particularly in low-income countries, with combination treatment being largely unaffordable [9–11, 13, 14, 18]. This result was shown by recent and earlier studies. In the studies that were reviewed, less than 1–53.5 days wages were required to purchase one month supply of CV medicines. Failure to meet affordability was 9–75%. In the public sector one month supply of 1 generic CVD medicine cost on average 2.0 days’ wages, and 1 originator brand CVD costs on average 8.3 days’ wages for the lowest paid government worker in a study published in 2010 [11]. Some recent multinational studies show that 1–13 times the wage of the lowest paid government worker (LPGW) is required to purchase one month supply of CV medicines [9, 13, 19]. Some national studies however, show that up to about 53 times the wage of the LPGW is required to purchase CV medicines [17]. Five studies reporting affordability with the number of days’ wages of the LPGW in public health facilities showed that, on average 1.6 days’ wages of the LPGW was required to purchase generics in the public sector [9, 11, 13, 18, 19]. In a study using a threshold of 20% of a household’s capacity to pay, combination of four CV drugs was unaffordable to 33% and 60% of households in lower-middle and low-income countries respectively [21]. In a recent study, 75% and 24% of households could not afford two anti-hypertensive drugs and a statin in low-income and-middle income countries respectively [8]. Affordability is frequently lower in the private sector although availability is higher. For CV medicines meeting both affordability and availability targets, findings show low achievement [9, 14]. In a recent multinational study, CV medicine affordability and availability target was met in 37.5% of adults [14]. Table 1 shows affordability and availability of cardiovascular medicines in LMICs.

**Table 1:** Availability and Affordability of cardiovascular medicines

Author and year of publication	Study design	Study setting	Sample size	Type of CV drug (Anti- hypertensive, statins and anti-platelets) studied	Number of CV drugs	Summary of findings
Attaei et al., 2017 [8]	Survey of local pharmacies and households Analysis of The Prospective Urban Rural Epi-demiological (PURE) study: 1st phase	Community pharmacies and households in High income, middle income and low- income countries	16 low- and middle-income countries; 511 communities	Anti-hypertensive and statin only	9	Availability Proportion of communities with four drug classes available was 76% in India (68 of 90), 71% in UMICs (90 of 126), 47% LMICs (107 of 227), and 13% in LICs (nine out of 68) Affordability Proportion of households unable to afford two BP-lowering medicines was 31% in LICs (1069 of 3479 households) and 9% in MICs (5602 of 65 471) Inability to afford two BP medicines plus a statin was 75% in LICs, 22% in lower middle and 26% in UMICs
Ewen et al., 2017 [9]	A facility-based survey using the WHO/HAI methodology	healthcare facilities and pharmacies in low-, lower-middle and upper-middle income countries	2161 outlets in 30 countries	All 3 CV drugs	15	Availability In LICs, median generic availability was 40.2% and 59.1% in the public and private sectors, respectively. Overall generic availability was 54.6% and 65.7% in lower-middle income countries and 56.7% and 76.7% in UMICs in the public and private sectors, respectively Median availability of any product type (originator brands and generics) was 43.3% and 66.7% in the public and private sectors of LICs, respectively. In lower-middle income countries. It was 57.6% and 68.6% in the public and private sectors respectively. In UMICs, median availability was 60.2% in the public sector and 90.0% in the private sector Affordability No more than 1 day’s wage was needed to buy LPG. 1.9 to 3.5 days’ wage was needed to buy originator in public sector Percentage of LPG meeting affordability and availability in the public sector was 11.9%, 33.8% and 34.5% in LI, LMI and UMI countries respectively. It was 22%, 36.7% and 50.9% in the private sector of LI, LMI and UMI countries
Harrison et al., 2021 [10]	Survey of health facilities and patients	Outpatient department of a teaching hospital and two community pharmacies in Ghana	Two health facilities; 304 outpatients; 3 health facilities	Anti-hypertensive drugs only	10	Affordability 74.7% had affordability for four drugs at the public hospital whiles 59.5% of patients could afford 4 drugs at the private pharmacy. 86.2% of patients could afford one BP lowering medicine out-of-pocket at the hospital whiles it was 81.9% at the private pharmacy Availability 15% of patients always obtained all their prescribed anti-hypertensive medication (continuous access) from the hospital pharmacy. Availability was 60%
Van Mourik et al., 2010 [11]	Analysis of data obtained surveys using the WHO/HAI methodology	public and the private health facilities in LI, LMI, UMI and HI countries	45 surveys from 36 countries	Anti-hypertensive medication	5	Availability of CV medicines in LMICs was poor (14.4% to 20.8% for generics in the public sector and 52.3% to 60.1% for generics in the private sector in LMICs). Originator brand availability in public sector was 0.6% to 21.4%) Affordability Average cost in public sector was 2.0 (LPG) and 8.3 (OB) day's wages to purchase one CV medicine for a month. Average affordability was better in the private sector (1.8- and 5.3-day’s wages for the LPG and OB) but the private sector was less affordable if countries were matched
Husain et al., 2020 [13]	Survey of lowest priced generics and originator brands using WHO/HAI survey method; secondary data aggregated at the national level	Public and private health facilities in low-, lower-middle, upper middle- and high-income countries	84 surveys in 59 countries	Anti-hypertensive drugs and statins	9	Availability Average availability was 54% in low- and lower-middle-income countries and 60% in high- and upper-middle-income countries (generic availability—61%; brand availability—41%). Availability of generics was 55% and 67% in public and private sectors respectively Affordability Average costs of 1 month’s antihypertensive medications were 6.0 days’ wage for brand medicine and 1.8 days’ wage for generics. Affordability was lower in low- and lower-middle-income countries than high- and upper-middle-income countries for both brand and generic medication
Chow et al., 2020 [14]	Survey of local pharmacies and households; Analysis of data from The Prospective Urban Rural Epi-demiological (PURE) study	Community pharmacies and households in High income, middle income and low-income countries	17 low- and middle- income countries; 592 communities	All 3 CV drugs	10	Availability CV medicines were not available in 6.9% to 55.8% of communities. Availability of all 3 types of CV drugs (antihypertensive, statin, antiplatelet) in communities in low- and middle-income countries was 50% to 87.2%) Affordability Percentage of High-Risk Adults with All 3 types available and affordable was 37.5% (34,974/93200) at 20% affordability
Ibrahim et al., 2021 [15]	survey using the World Health Organization/Health Action International (WHO/HAI) guideline	Retail medicine outlets in Public Hospitals, Private Hospitals, Private Pharmacies, Public Healthcare Centers	30 facilities	All 3 CV drugs	11	Availability of CV medicines was 27.2% in public hospitals, 6.1% in public health centres and 77.6% for private pharmacies
Kibirige et al., 2017 [17]	WHO and HAI standardised methods-based questionnaire	public hospitals, private hospitals and private pharmacies in 4 regions of Uganda	145 facilities	All 3 CV drugs	28	Availability of CV medicines was 39.4% and 74.1% in the public and private facilities respectively Affordability Less than 1 to 53.5 days’ wages were needed to purchase lowest priced generic drugs in both private hospitals and pharmacies
Argawal et al., 2022 [18]	A cross sectional survey using WHO/HAI methodology	Public and private facilities in low- and middle- income countries	53 low- and middle-income countries	Anti-hypertensive medication	6	Availability was 19.03% to 76.9% for LPG in the public sector. Availability of LPG in the private sector was 41.1% to 80.49% Affordability 0.2 to 3.11 days wages were needed to purchase LPG in the public sector. 0.45 to 3.4 days wages were needed to purchase LPG in the private sector. 2.85 to 7.32 days wages was required to purchase originator drug in public sector
Dzudie et al., 2020 [19]	Survey using the WHO/HAI methodology	public, confessional, private facility medicine outlets, and community pharmacies in Cameroon	63 medicine outlets	All 3 CV drugs	22	Availability was an average of 16.1% in public facility outlets and 16.4% in community pharmacies, being higher in urban and semi-urban compared to rural outlets. Affordability Beta blockers, ACE inhibitors and statins required 2–5 days and 6–13 days wages respectively for one month of chronic treatment. Aspirin, digoxin, furosemide, HCTZ and nifedipine were affordable (cost a day’s wage or less)
Khatib et al., 2016 [20]	Analysis of the Prospective Urban Rural Epi-demiological (PURE) study; survey	Pharmacies and community households	596 communities in 18 countries	All 3 CV drugs	4 classes	Availability CV medicines were available in 80% of urban and 73% of rural communities in UMIC, 62% of urban and 37% of rural communities in LMIC, 25% of urban and 3% of rural communities in LIC (excluding India) Affordability CV medicines were unaffordable for 25% of households in UMIC, 33% of LMIC, 60% of LIC (excluding India

In LMICs, procurement prices are higher than the international reference price (IRP). Compared with the international reference price, the government procurement prices, on average, were 17 times higher for brand medicines, 4.5 times higher for the lowest-priced generic and 21.7 times higher for the brand medicines in the LLMICs in one recent study [13]. Patient prices vary largely from procurement prices. In the public sector of LLMICs, a recent study showed that while procurement price for generic medicines was 4.5 times the IRP, the patient price for the same generics is 11.2 times the IRP [13]. Patient prices have been found to be higher in LLMICs compared to UMICs. In a study by Husain et al., patient prices in LLMICs was higher than the average of 80.3 and 16.7 times the IRP for branded drugs and generic CV medicines respectively [13]. Patient prices in the private sector are significantly higher than the public sector. Husain et al. reported LLMIC Median Price Ratios (MPR) for the private and public sectors as 95.2 and 46.4 respectively for branded medicines [13].

Studies on accessibility showed that inadequate presence of healthcare workers and long distance to health facilities has been reported as a challenge to accessibility of medicines and healthcare in LMIC [21, 24]. In sub-Saharan Africa one recent study reported that patients travel 25.29 ± 14.72 and 53.29 ± 17.86 min to reach health facilities for antihypertensive medicines, while another reported rural inhabitants travel between 1 to 2.5 h to reach health facilities [21, 22]. A study in LMIC showed that 5–35% of patients with chronic diseases travel more than 15 min to visit health facilities [24].

Acceptability of CV medicines in some countries is low, yielding low rates of treatments although medicines are available. Adherence to treatment guidelines is low in many LMICs [23, 25]. One study of patients with heart failure in a rural Haiti health facility where medicines were available and supplied free to patients is a typical example of provider acceptability barriers. During discharge, only 21% of heart failure patients were treated with the evidence-based combination cardiovascular medicines [25]. Non adherence to cardiovascular medicines is significant. Patients’ beliefs that medication are unnecessary and that medication will cause side effects contribute to non-adherence [26, 27].

The presence of substandard and falsified medicines is a challenge in many LMICs, especially Africa, and regulatory capacity is inadequate. A recent study in Africa reported 16.3% poor quality cardiovascular medicines [28]. Amlodipine and captopril have been reported with a higher poor quality [28] Heart medicines are among the 5th most frequently reported (5%) substandard class of medicines in the WHO monitoring system [29].

### Measures for improving access

While significant challenges in access to cardiovascular medicines faces LMICs, opportunities exist for improvement. The WHO and other authors have published policies and measures that could address challenges in access to CV medicines. Efficient forecasting and procurement, increased public financing, abolishing taxes, policies to improve generic use, regulating mark-ups, and ensuring that CV medicines are added to the EML are measures for improving availability and affordability. Other measures are social health insurance and provision of incentives, passing on low procurement prices to the private sector, price negotiation, promoting market competition, quality monitoring, prioritizing essential medicines budgets and good health system governance [7, 9, 12, 29, 31]. Table 2 summarizes steps to improve access to CV medicines.

**Table 2:** Measures for improving access to medicines

Access measure	Interventions to improve access	Wirtz et al. [7]	Ewen et al. [9]	Mendis et al. [12]	Antignac et al. [28]	Acosta et al. [31]	WHO [32]
Availability	Efficient projections, procurement, transparency, good governance; Improving the selection process for medicines Increase public financing for cardiovascular medicines Strong generic policy: Create incentives in the public and private sectors to make low-price, quality-assured medicines available Public health funding Consideration of Market-related factors (high demand, voluntary withdrawal, a shift in clinical practice, loss of interest in the market and changing the location of production facilities) Improve manufacturing processes Address ethical issues (such as regulatory problems) Consider Supply stage of medicines, market competition, and possible therapeutic substitutes	**√**				√	
Affordability	Abolish taxes and duties on essential medicines and control mark-ups Improve market competition (price information, price competition); Pooled procurements in specific contexts may work Scale up insurance programs (prepayment schemes; universal health coverage; social protection) Provide incentives to increase local production with fair pricing	√					
Accessibility	Increase operational hours Decrease waiting times Increase perceived quality of care, eg, patient satisfaction surveys to monitor changes and identify gaps and needs	√					
Acceptability	Rational use of medicines (National treatment guidelines, EML) Provide Fixed Dose Combination (FDC) medications (polypill)	√					
Quality of medicines	Provide incentives to businesses to invest in quality medicines Follow good procurement practices Establish systems to verify authenticity; strengthen regulation Continuous monitoring and public awareness as well as national and international scrutiny as measures against substandard and falsified medicines	√			√		
Availability and affordability	Accelerated and lower-cost registration procedures for generics Efficient government procurement; Pass on low procurement prices Adequate forecasting, adequate and sustainable financing, efficient distribution system, Removing taxes and tariffs on essential medicines Regulating mark-ups in the supply chain Institute mandatory prescribing by the medicine’s International Nonproprietary Name (INN) Promote generic substitution Incentives for dispensing of lower-priced generics (regressive mark-ups) Promote the use of lower-priced generics to health professionals and the public; quality-assurance, publicly available quality testing Introduce market competition Improving governance and management efficiency Assess local supply options Prioritize the essential medicines drugs budget Purchase low-priced quality generics Provide essential chronic disease medicines through the private sector at public sector procurement prices Therapeutic substitution Direct price negotiations; price transparency; Set generic prices from procurement prices if regulating price Differential pricing (tax exemption for government facilities) Establish a national Essential Medicines List Pooled procurement of government institutions, tendering		√	√			√

√ means the access measures were reported by the author/s

## Discussion

Our review shows that in many low- and middle- income countries, affordability and availability of CV medicines is low. Between economies and within countries there are equity gaps in access to CV medicines in the health system. In settings or facilities where medicines are available, they are often not affordable. In setting or facilities where medicines are affordable, they are often not available. These findings suggest that reducing mortality from CV disease in LMICs may remain a challenge. 80% of CVD deaths occur in low- and middle- income countries (LMICs) and the mortality burden from CV disease has been predicted to rise in LMICs [33–36]. Limited access to potentially lifesaving medications in LMICs makes guideline-based practice in the treatment and prevention of CV disease challenging [37].

Barriers to access to CV medicines in LMICs have been discussed in literature. At the policy level, limited national funding as a result of competing health policy priorities, slow inclusion of CV medicines into the essential medicines list (EML), as well as structural barriers limit access to CV medicines [38, 39]. In LMICs, lack of adequate public financing of essential cardiovascular medicines limits availability of these medicines. Although social health insurance has been implemented in some LMICs, insufficient funding leads to poor availability of medicines which are supplied free of charge. Patent laws have also been suggested as barriers to access to CV medicines in LMICs.

The rate limiting step in access to CV medicines lies within the health system’s supply chain. In the public sector of LMICs poor availability of CV medicines results in patients accessing medicines from private facilities where medicines are often not affordable [12, 40]. High prices of medicines lead to a situation where patients in many low and-middle income countries may have to spend catastrophic proportions of their income on medicines. Although medicines are supplied free in some settings, availability is sometimes poor in settings where medicines are supplied free [9]. Where medicines are supplied on health insurance, coverage may be low. In LMICs, there is complex supply chain design as multiple tiers of stock management occur before medicines can reach patients taking the medication [41]. Lack of accountability in the supply chain and low density of health care workers have also been identified as drivers of limited access to CV medicines [42]. A review by Almuzaini et al. shows that systemic deficiencies and inefficiencies in regulation and distribution of medicines have contributed to the burden of counterfeit medicine in LMICs [42].

Our study highlights significant disparities in access to CV medicines between the private and public sectors, countries and income levels. In the private sector, although CV medicines are more available, they are less affordable, suggesting that both sectors are critical players in access to CV medicines. The disparities in access between urban and rural communities also suggests that policy measures to improve access to CV medicines should consider factors influencing this disparity. Our findings show that low- income economies have the heaviest challenge, suggesting a need for urgent action as high cardiovascular morbidity and mortality are likely to compound the economic challenges of these economics. Disparities in findings should however, take into account differences in study characteristics. Measurements of affordability, for example, were performed with different methods. A review of availability and affordability of CV medicines by Lotfizadeh et al. showed similar findings [43].

Inadequate accessibility of CV disease treatment probably reflects inefficient transportation systems, infrastructural inadequacies and low density of providers of care in LMICs. In low-income countries especially, there is insufficient number of healthcare facilities, and people need to travel a long distance to access healthcare facilities [21, 22, 24]. Non-adherence to local guidelines as well as patient non adherence contributes to low acceptability [44]. In some LMICs, physicians’ preference for specific types of brands of CV medication creates a demand for medicines not listed in the essential medicines list. Complex medical treatment contributes to low medication adherence as many CV disease patients receive multiple medicines.

Measures which are setting-, health system- and facility-contextualised are important strategies for improving access to cardiovascular medicines [9]. Some of the policy measures are increased public financing of CV medicines, efficient procurement, increased health insurance coverage, strong generic policy, market competition, removing taxes, passing on low procurement prices to the private sector, regulating mark-ups and good governance which pays attention to system design and effective oversight [7, 9, 12, 31]. Public financing can be used to boost local manufacture of CV medicines. Such a policy has significant relevance in LMICs since many local manufacturers of medicines have limited funding. Increasing public financing is key to ensuring efficient reimbursement for supplies of health insurance medicines. In LMICs, delays in reimbursement for supplies of medicines at service delivery points is an important source of financial constraint to providers, and this contributes to CV medicine availability gaps. Investment in the private sector can boost availability. Efficient procurement of CV medicines can contribute to availability and affordability. This may be achieved by procuring lower-priced generic CV medicines, negotiating prices with pharmaceutical suppliers, using pooled procurement at the national level as well as competitive tendering. Tax exemptions can encourage local manufacture, lower prices from pharmaceutical wholesalers and subsequently lower retail costs. Tax exemptions for public facilities could also lower prices of medicines in the public sector.

A strong generic policy that ensures accelerated lower-cost registration procedures for generic CV medicines and mandatory prescribing by the drug’s International Nonproprietary Name (INN) as well as a policy that promotes generic substitution and provides incentives for dispensing lower-priced generic CV medicines can contribute to both availability and affordability. A study by Attaei et al. has shown that in India where there is a large generic market, availability of CV medicines is comparable to high income countries [8]. Price transparency and competition are important for market competition. Generic competition can lower prices of CV medicines. The association between generic competition and lower prices has been reported in literature [45].

Health insurance can improve access to CV medicines in LMICs by preventing catastrophic health expenditures and making CV medicines affordable (free of charge) [46]. In settings where medicines are not supplied free of charge, government can procure and make CV medicines available to the private sector at low procurement prices. The complex supply chain in LMICs contributes to mark-ups and high patient prices. The WHO recommends mark-up regulation across the supply chain. This should however, be carefully considered. Other pricing policies should be considered alongside mark-up regulation [47]. Also, regressive mark-up, where rate of mark-up decreases as the price increases (as opposed to a fixed percentage mark-up for all prices) is a better system. Remuneration and mark-up regulation can be used as incentives for supplying generic medicines and those reimbursed or supplied to health insurance patients [47].

Other measures for improving access are therapeutic substitution, ensuring inclusion of CV in the EML, efficient distribution, prioritizing the essential CV medicines budget and ensuring continuous quality monitoring. Therapeutic substitution policies enable alternative CV medicines to be used and can contribute to continuation of treatment and lower drug costs [48]. Formulary managers and Drug and Therapeutic Committees (DTC) can list therapeutic alternatives in the formulary and institute a therapeutic exchange policy.

Making CV medicines an integral part of the national EML is a key step towards achieving their availability and affordability in the health system [49]. The WHO model EML is meant to be adopted by countries to prioritize essential medicines such as CV drugs, and ensure availability [5]. Studies have reported the link between inclusion of medicines on the EML and availability and affordability of the medicines [49, 50]. Twagirumukiza et al. showed in their study that in Sub-Saharan Africa, antihypertensive drugs found in the EML had lower prices than those that were not found in the list [50]. A multinational study reported 61.5% availability of medicines included in the EML compared with 27.3% availability for non EML medicines [49]. The selection of medicines for inclusion in formularies and procurement needs consideration and prioritisation of medicines in the EML.

An efficient supply chain helps in ensuring better forecasting of medicine need with consequent decrease in stock-outs or overstocking. Efficient distribution systems can be achieved through the use of network information systems to ensure availability of demand and supply data. By reducing the number of steps between central medical stores and patient distribution centers, and improving transportation, access to CV medicines can improve [51]. In Zambia, Vledder et al. in a randomised study showed that a distribution model that uses a direct delivery of health supplies from the central medical stores to various healthcare facilities significantly lowered the incidence of essential medicine stock-outs compared to a multi-level distribution system [51]. Technology can improve efficiency in fragmented supply chains. Mobile phone technology has shown benefit in Kenya and Ghana. Consumption data can be transmitted by clinic workers to the central stores to inform restocking and future procurement. In Ghana, the Early Warning System, an SMS/web-based system had a positive impact on essential tracer medicine stock status information flow and visibility, and ensured availability at service delivery points [52].

Continuous quality monitoring can improve the availability of low priced quality generic CV medicines. Publicly available quality testing is recommended. Secure tracking and tracing systems can contribute to ensuring that suppliers of counterfeit medicine do not have room to distribute their medicines through legitimate pharmaceutical supply chains.

To complement policy efforts of governments, various stakeholders have a role to play to address challenges in access to CV medicines. Healthcare facility managers need to institute policies that encourage generic prescribing and therapeutic substitution and ensure efficient procurement. Healthcare managers also need to ensure the procurement of low-priced quality generics. Public central medical store managers, service delivery point stores/pharmacy managers and pharmaceutical supply companies have a duty to ensure efficient distribution and stock management. Regulators have to ensure continuous CV medicine quality monitoring. Healthcare facility managers need to ensure that, selection of CV medicines for inclusion in formularies as well as their procurement give priority to drugs on the EML. Prescribers and pharmacists have a duty to promote the use of generic CV medicines.

This review has some limitations. It did not assess the risk of bias in the studies that were analysed. The study included only English language articles. Literature which was not found in PubMed and Google Scholar was outside the scope of this study. Since articles in other databases may meet the inclusion criteria, the findings of this study reflect the literature scope that was searched for this study. It is however noteworthy that some articles found in PubMed may also be found in other databases. Google scholar has limitations. For eg. While a search can produce a high number of articles, precision is lower than with other databases. Also, the variation of search term sequence may not produce same result.

## Conclusions

Significant gaps exist in access to cardiovascular medicines in LMICs, and in many LLMICs access to cardiovascular medicines is low. This review shows that meeting both affordability and availability targets is particularly a challenge. To improve access, reduce the burden of CVD and achieve the Global Action Plan on non-communicable diseases in these countries, policy interventions must be urgently instituted. Governments in LMICs need to increase public financing for CV medicines; they need to create incentives such as tax exemptions in the public and private sectors. Government policy makers also need to adopt a strong generic policy and efficient medicine distribution models, promote market competition and ensure efficient government procurement. Other important policy measures are prioritizing the essential medicines budget and adding CV medicines to the EML, regulating mark-ups, and increasing health insurance coverage. At the national level, access to essential CV medicines needs to be monitored to inform contextualised policies. Healthcare and procurement managers, prescribers, Drug formulary managers and pharmacists need to support efforts to improve access to CV medicines.

## Data Availability

The datasets used for analysis of this study are available from the corresponding author upon reasonable request.

## Abbreviations

ACE: Angiotensin converting enzyme
CV: Cardiovascular
CVD: Cardiovascular disease
DTC: Drugs and therapeutics committee
EML: Essential medicines list
HAI: Health action international
HCTZ: Hydrochlorothiazide
LICs: Low income countries
LI: Low income
LLMICs: Low- and lower middle- income countries
LMI: Lower middle income
LMICs: Low and middle income countries
LPG: Lowest priced generic
LPGW: Lowest paid government worker
MPR: Mean price ratios
NCDs: Non communicable disease
OB: Originator brand
UMI: Upper-middle income
UMICs: Upper-middle income countries
WHO: World Health Organization
